# Patient-level data linkage across ambulance services and acute trusts: assessing the potential for improving patient care

**DOI:** 10.23889/ijpds.v1i1.290

**Published:** 2017-04-19

**Authors:** Sophie Clark, Michael Damiani, Holly Dorning, Mary Halter, Alison Porter

**Affiliations:** The London Ambulance Service; The Nuffield Trust; Kingston and St. Georges, The University of London; Swansea University

## Objectives

The potential of linked healthcare data to support improvements to the care quality, efficiency and service planning is recognised by the Department of Health and NHS England. UK ambulance services however, have little information regarding the outcome of patients as there is no routine data sharing. An urban ambulance service has previously piloted linked data with one acute trust. The Pre-Hospital Emergency Department Data Linking Project (PHED Data) aims to assess the potential opportunities for and challenges to routinely linking data for several sites. It aims to define what indicator sets can be developed from these linked data, with a view to informing commissioning and improvement of healthcare delivery. 

## Approach

The project is a two-year mixed-methods observational study, funded by the Health Foundation, working with six acute trusts of various size and CQC-derived performance, to carry out six work packages.

Work package one uses liaison activities with trust senior staff to negotiate information sharing agreements, and a learning log to enable an economic assessment of the set-up costs.

Work packages two to five analyse ambulance response time, referrals from healthcare professionals, ED mortality, and frequent ED diagnoses, respectively, exploring relationships with ED outcomes, quantitatively. Qualitative analysis will explore, with staff groups, how the findings might influence commissioning and pre-hospital care. Work package six will examine commissioning decisions and patient care, through interviews with commissioners and performance managers. 

## Results

In work package one, we have successfully negotiated overall research ethics and governance approval, involving in-depth discussion about definitions of identifiable patient data, and protecting against potential re-identification. We have recruited six acute trusts, comprising 13 hospital sites, for whom equivalent data is available. We have been working for six months in liaison with these Trusts to deliver information sharing agreements (three are currently approved) and data transfer, working with research governance, information governance and clinical staff. The processes have varied, with intra-Trust co-dependencies introducing delays and non-linear processes being common.

For work packages two to five, some data analysis will be available for presentation at the conference. 

## Conclusion

This project has the potential to shed light on the practicalities of data linkage for health service providers who face similar challenges with patient data held in multiple organisations.

The study also anticipates being able to recommend quality improvement to support the development of new pathways in pre-hospital care. 

